# Commentary: Serum Vitamin D Levels Are Associated With Increased COVID-19 Severity and Mortality Independent of Whole-Body and Visceral Adiposity

**DOI:** 10.3389/fnut.2022.885204

**Published:** 2022-03-28

**Authors:** Marijn M. Speeckaert, Joris R. Delanghe

**Affiliations:** ^1^Department of Nephrology, Ghent University Hospital, Ghent, Belgium; ^2^Research Foundation-Flanders (FWO), Brussels, Belgium; ^3^Department of Diagnostic Sciences, Ghent University, Ghent, Belgium

**Keywords:** COVID-19, vitamin D (PubChem CID: 5280793) 2, vitamin D binding protein (DBP), thrombosis, inflammation

## Introduction

With interest, we read the paper of Vanegas-Cedillo et al. (1), which showed an independent association between vitamin D deficiency and COVID-19 mortality, after adjustment for BMI and epicardial fat. Vitamin D deficiency may contribute to a pro-thrombotic and pro-inflammatory state in COVID-19. Although some confounders were taken into account, we would like to illustrate the potential influence of vitamin D binding protein (DBP) on the reported findings.

## Vitamin D Binding Protein and Its Polymorphisms

Vitamin D and its metabolites are mostly (85%) carried by DBP and to a lesser extent (10–15%) by albumin in the bloodstream, with <1% present in a free unbound form. DBP is one of the most polymorphic proteins in humans, with three common alleles [DBP1S (slow), DBP1F (fast), and DBP2] and > 120 unique racial variations. DBP1S [rs7041-G (ASP), rs4588-C (Thr)], DBP1F [rs7041-T (ASP), rs4588-C (Thr)], and DBP2 2 [rs7041-T (ASP), rs4588-A (Lys)] are determined by two single-nucleotide polymorphisms (SNPs, rs4588, and rs7041) in the *DBP* gene (2). DBP phenotypes influence plasma concentrations of 25-hydroxyvitamin D (25OHD), 1,25-dihydroxyvitamin D [1,25(OH)_2_D], and DBP. Patients with the DBP1-1 phenotype have the highest concentrations, while those with the DBP2-1 and DBP2-2 phenotypes have intermediate and lowest levels, respectively. The serum DBP level shows a positive correlation with the 1,25(OH)_2_D concentration (3). A genome-wide meta-analysis discovered other SNPs that impact the 25OHD concentration, such as rs2282679 of the *DBP* gene, which is a near-perfect proxy for rs4588. rs2282679-A and rs4588-C are frequently co-inherited, whereas rs4588-C is frequently co-inherited with rs2282679-A. Vitamin D levels are lower in rs2282679-C/C carriers than in subjects with only one rs2282679-C-allele, who in turn have lower vitamin D levels than rs2282679-A/A individuals (4). In COVID-19 patients with acute respiratory distress syndrome (ARDS), measuring DBP levels may be especially important in determining 25OHD concentrations, because DBP is a negative acute-phase protein, with levels plummeting by nearly a third in patients with ARDS (5).

## Vitamin D Binding Protein and COVID-19

The potential link between DBP phenotypes and COVID-19 has been investigated by our research group (6). The prevalence and mortality owing to a severe acute respiratory syndrome coronavirus 2 (SARS-CoV-2) infection were negatively correlated with the DBP1 allele frequency (a combination of DBP1S and DBP1F). This finding could be partly explained by the potential protective effects of vitamin D, as described in the paper of Vanegas-Cedillo et al. (1). In another study, polymorphisms in the *DBP* gene were also related to the infection severity of COVID-19 (7). The vitamin D total risk score (*DHCR7*; *CYP2R1*; *DBP*; *CYP24A1*; *AMDHD1*; *SEC23A*) was associated with the serum 25OHD concentration. The metabolism score (*DBP*; *CYP24A1*) correlated positively with patient outcomes in COVID-19. The majority of the association might be explained by rs2282679 in the *DBP* gene. Aside from transporting vitamin D, DBP may also fulfill other functions in this process.

## Discussion

Because only around 4% of DBP is conjugated with vitamin D metabolites, this plasma protein performs a variety of additional tasks, including actin scavenging, fatty acid-binding, endotoxin transporting, and chemotactic cofactor activating (8). Low vitamin D concentrations may contribute to a pro-thrombotic and pro-inflammatory state, which is observed in COVID-19 patients (1). In both pathophysiological pathways, DBP might play a key role. Thrombosis is a serious complication and a critical aspect of COVID-19 progression. Local tissue injury and/or cell death result in the release of monomeric globular actin (G-actin), which escapes normal intracellular regulatory systems and polymerizes to form actin filaments (F-actin). This common feature increases blood viscosity, triggers disseminated intravascular coagulation and multiple organ dysfunction syndrome (9). Extracellular F-actin filaments disrupt the coagulation and fibrinolytic systems, causing blockage and damage to the microcirculation (especially in the lung) (10). The extracellular actin scavenging system, which consists of gelsolin and DBP, cleaves actin and prevents repolymerization to counteract the procoagulant effects. Gelsolin severs and depolymerizes actin filaments, whereas DBP can impede new filament production and sequester actin. The actin scavenging system plays a crucial role in respiratory failure and severe sepsis. Actin-induced depletion of plasma DBP to concentrations <3.5 μM (200 μg/mL) is an effective but indirect marker of tissue injury and has been associated with higher APACHE II and SOFA scores, and with poor overall survival (9). This might also explain why DBP2 allele carriers have a higher COVID-19 mortality rate while having lower vitamin D metabolite and DBP concentrations (6). Furthermore, aspirin treatment resulted in considerably greater DBP concentrations post-aspirin treatment compared to pre-treatment in cerebral thrombotic patients, but the reverse event was observed for actin. Aspirin, through the activities of DBP and other DBP-related proteins, may inhibit platelet aggregation and thrombosis (11).

The inflammatory cytokine storms connected to severe COVID-19 cases and the virus's rapid transmission might be explained by hyperactivation of chemotaxis and immune cell trafficking, as well as enhanced fatty acid synthesis (12). DBP has no direct anti-inflammatory properties. DBP is a direct positive regulator of neutrophil chemotaxis, works as a neutralizer of endogenous inhibitors of chemotaxis, and is involved in fatty acid-binding (8). The effect of vitamin D on a particular T cell response *in vivo* is complex and presumably depends on a variety of parameters in addition to the 25OHD concentration, such as the local concentration and rate of DBP degradation, as well as the distinct DBP phenotypes (13). Neutrophil elastase may play a key part in the C5a co-chemotactic pathway, regulating the quantity of DBP bound to cells, by shedding its binding site (14). Human neutrophils lose their chemotactic cofactor function when 1,25(OH)_2_D binds to DBP, and oleic acid is one of the tonic inhibitors of chemotaxis in human plasma. Finally, DBP binds fatty acids, particularly polyunsaturated fatty acids, as well as the membranes and chondroitin sulfated proteoglycans of leukocytes (15). Polyunsaturated fatty acids compete with vitamin D metabolites, decreasing DBP's apparent affinity for 25OHD and 1,25(OH)_2_D in particular (16).

In conclusion, while examining the potential relationship between vitamin D deficiency and COVID-19, we propose taking into account DBP and its polymorphisms.

## Author Contributions

MS wrote the first draft of the manuscript. JD edited and revised the manuscript for important content. Both authors reread, edited, and approved the final version of the manuscript for submission.

## Conflict of Interest

The authors declare that the research was conducted in the absence of any commercial or financial relationships that could be construed as a potential conflict of interest.

## Publisher's Note

All claims expressed in this article are solely those of the authors and do not necessarily represent those of their affiliated organizations, or those of the publisher, the editors and the reviewers. Any product that may be evaluated in this article, or claim that may be made by its manufacturer, is not guaranteed or endorsed by the publisher.
